# Epigenetic Regulation of Depot-Specific Gene Expression in Adipose Tissue

**DOI:** 10.1371/journal.pone.0082516

**Published:** 2013-12-05

**Authors:** Sandra Gehrke, Bodo Brueckner, Andreas Schepky, Johannes Klein, Alexander Iwen, Thomas C. G. Bosch, Horst Wenck, Marc Winnefeld, Sabine Hagemann

**Affiliations:** 1 Beiersdorf AG, Research and Development, Hamburg, Germany; 2 Heidelberg, Germany; 3 University Medical Center Schleswig-Holstein, Department of Medicine I, Luebeck, Germany; 4 Christian-Albrechts-University, Zoological Institute, Kiel, Germany; Tohoku University, Japan

## Abstract

In humans, adipose tissue is distributed in subcutaneous abdominal and subcutaneous gluteal depots that comprise a variety of functional differences. Whereas energy storage in gluteal adipose tissue has been shown to mediate a protective effect, an increase of abdominal adipose tissue is associated with metabolic disorders. However, the molecular basis of depot-specific characteristics is not completely understood yet. Using array-based analyses of transcription profiles, we identified a specific set of genes that was differentially expressed between subcutaneous abdominal and gluteal adipose tissue. To investigate the role of epigenetic regulation in depot-specific gene expression, we additionally analyzed genome-wide DNA methylation patterns in abdominal and gluteal depots. By combining both data sets, we identified a highly significant set of depot-specifically expressed genes that appear to be epigenetically regulated. Interestingly, the majority of these genes form part of the homeobox gene family. Moreover, genes involved in fatty acid metabolism were also differentially expressed. Therefore we suppose that changes in gene expression profiles might account for depot-specific differences in lipid composition. Indeed, triglycerides and fatty acids of abdominal adipose tissue were more saturated compared to triglycerides and fatty acids in gluteal adipose tissue. Taken together, our results uncover clear differences between abdominal and gluteal adipose tissue on the gene expression and DNA methylation level as well as in fatty acid composition. Therefore, a detailed molecular characterization of adipose tissue depots will be essential to develop new treatment strategies for metabolic syndrome associated complications.

## Introduction

In humans, different adipose tissue depots are associated with different metabolic effects. 80-90% of total body fat is stored in subcutaneous adipose tissue depots, mainly in the abdominal, subscapular and gluteal areas [1]. Additionally, intra-abdominal depots which are associated with digestive organs and consist of visceral adipose tissue have been comprehensively analyzed [1]. It has been consistently shown that visceral fat mass is more strongly associated with metabolic syndrome than subcutaneous fat [2-4]. However, also excessive subcutaneous abdominal fat mass is strongly associated with insulin resistance and has been positively correlated with metabolic complications such as obesity [5]. In contrast, subcutaneous gluteal fat exerts a protective effect on metabolic disorders such as type 2 diabetes and atherosclerosis [6]. 

Adipocyte size and number varies as a function of obesity level and metabolic status. Adipose tissue expands by increasing the volume of preexisting adipocytes (hypertrophy) or by generating new adipocytes through adipogenesis (hyperplasia) with a positive correlation between fat mass and fat cell volume, which is strongly linked to metabolic complications of obesity [7]. Although both, preadipocytes from abdominal and preadipocytes from gluteal adipose tissue are considered as identical cell type, they have distinct expression profiles and distinct functions [8]. Thus, a detailed characterization of molecular differences between adipose tissue depots would provide an important basis to understand its heterogeneous characteristics and infer consequences for health and disease. 

In various studies, functional as well as gene expression differences between human subcutaneous and visceral adipose tissue have been discovered [9-11] whereas differences between abdominal and gluteal adipose tissue are characterized less extensively. Recently, differences in gene expression profiles have been documented in abdominal adipose tissue compared to gluteal adipose tissue [12-14]. However, in addition to gene expression analysis, the analysis of gene regulation will contribute to further our understanding of depot-specific differences in adipose tissue. One important mechanism for gene regulation is the epigenetic modification of DNA by methylation of CpG dinucleotides [15]. Epigenetic mechanisms play an important role in regulating developmental processes. After differentiation, each cell type is characterized by a specific methylation pattern that regulates cell-type specific gene expression [16]. Therefore, we hypothesized that epigenetic mechanisms might be functionally involved in specifying molecular differences between abdominal and gluteal adipose tissue. To address this question, we aimed at identifying epigenetically regulated genes that show depot-specific expression by combining methylation profiling and global gene expression analyses. To our knowledge, no genome-wide comparative DNA methylation analysis of different adipose tissue depots has been conducted yet.

In summary, our results indicate that each fat depot, subcutaneous abdominal and subcutaneous gluteal, has a different lipid composition and a unique developmental gene expression signature which is partially associated with differential DNA methylation. Considering these differences between adipose tissue depots will be important for understanding the development of obesity-associated diseases which might lead to new treatment options for metabolic-related disorders. 

## Methods and Procedures

### Ethics statement

The recommendations of the current version of the Declaration of Helsinki as well as the international guidelines (FDA Regulations, GEP and AWB guidelines) were observed. All participants in the contributing study provided written, informed consent. Biopsies were obtained through a clinical study approved by the Ethics Committee Luebeck (06-220).

### Recruitment and tissue specimens

Subcutaneous adipose tissue samples of 14 female volunteers aged 27 to 35 years (BMI Ø 22.5) were obtained from a clinical study. Exclusion criteria were significant medical illness, alcohol abuse, smoking, pregnancy and abnormal menstrual cycles. Subcutaneous adipose tissue samples from each volunteer were obtained by surgical biopsy techniques (about 2 – 4 cm^3^) under local anesthesia from abdominal and gluteal areas. Adipose tissue samples were washed in physiologic serum and immediately frozen in liquid nitrogen and stored at – 80° C for downstream analyses. Subcutaneous abdominal and gluteal adipose tissue samples from the same subject were used for analysis of gene expression (n = 10), DNA methylation (n = 6) and fatty acid composition (fatty acids n = 7, triglycerides n = 14). Detailed characteristics of all volunteers (age, BMI) and samples used for analyses (gene expression, DNA profiling, triglycerides and fatty acids) are provided in Figure S1.

### RNA and DNA isolation

Total RNA was isolated from surgical biopsies using the RNeasy Lipid Tissue Mini Kit (Qiagen, Hilden, Germany) according to the manufacturer`s instructions. Genomic DNA of surgical biopsy samples was isolated using the MasterPure^TM^DNA Purification Kit (Epicentre Biotechnologies) according to the manufacturer`s instructions. DNA and RNA were quantified using the NanoDrop ND-1000 instrument (Peqlab Biotechnologies, Wilmington, USA).

### Gene expression analysis

RNA of 10 subcutaneous abdominal and 10 subcutaneous gluteal fat depot samples (subcutaneous abdominal and gluteal samples derived from the same subject) was quality-controlled and processed by Miltenyi Biotech GmbH (Bergisch Gladbach, Germany) and loaded on single-color Whole Human Genome 4x44K microarrays (G4112F) from Agilent Technologies. Agilent Feature Extraction Software (AFE) was used to read out the microarray data sets. Next, data were imported into the R statistical software [17], quality-controlled and background-corrected using the Bioconductor Agi4x44PreProcess package [18]. All arrays were quantile-normalized and log_2_-transformed. Except of one abdominal sample (sample 45_abdominal) all samples showed good quality and could be used for further analysis. Unsupervised hierarchical clustering (euclidean distance) was performed using the function hclust of the R statistical package. To identify genes differentially expressed in abdominal and gluteal fat samples, the limma package [19] for the R statistical software was used to compute a moderated t- test. Raw p values were adjusted using the Benjamini-Hochberg procedure [20] and p ≤ 0.01 was used as statistical significant threshold.

### Quantitative RT-PCR

TaqMan Gene Expression Assays (Applied Biosystems, FosterCity, USA) were used to confirm the differential expression detected by genome-wide analysis. For technical and independent validation, samples used for microarray analyses (n = 5) and three independent samples were reverse-transcribed into cDNA and PCR reactions were carried out in triplicates as recommended by the manufacturer. A detailed overview of the samples used for validation is shown in Figure S1. Quantitative RT-PCR of PhospholipaseA2, group2 (*PLA2G2A*, Assay ID HS00179898_m1), Fibroblast growth factor 10 (*FGF10*, Assay ID Hs00610298_m1), Insulin-like growth factor binding protein 5 (*IGFBP5*, Assay ID Hs00181213_m1), homeobox B6 (*HOXB6*, Assay ID Hs00980016_m1), homeobox D8 (*HOXD8*, Assay ID Hs00251905_m1), homeobox C10 (*HOXC10*, Assay ID Hs00213579_m1), homeobox C12 (*HOXC12*, Assay ID Hs00545229) was performed using the Applied Biosystems 7900HT Fast Real Time PCR System. C_T_ values were calculated by the RQ Manager software 1.2. Gene expression levels in abdominal tissue were normalized to expression levels in gluteal tissue and *GAPDH* (Assay ID Hs99999905_m1) was used as a reference. To quantify the results obtained by real-time RT-PCR we used a comparative Ct method, also known as the 2^-ΔΔCt^ method. This involves comparing the PCR signals (Ct values) of samples of interest with a reference or control such an untreated control (ΔΔCt). The Ct values of both (sample and reference) were normalized to an appropriate endogenous housekeeping gene (ΔCt): ΔΔCt = ΔCt [sample] – ΔCt [reference]. Furthermore, unpaired t-tests were performed with a significance level of 0.05 (alpha).

### DNA methylation profiling

DNA methylation profiles of 6 abdominal adipose tissue samples and 6 gluteal adipose tissue samples (subcutaneous abdominal and gluteal samples derived from the same subject) were generated using Infinium methylation 450K BeadChips from Illumina (Illumina, San Diego, USA), which enquire 485,577 cytosine positions of the human genome distributed across different genomic regions, e.g. the promoter, 5`UTR, first exon, gene body and 3`UTR as well as intergenic regions. Bisulfite treatment and conversion of DNA for methylation analysis was carried out using the EZ-96 DNA Methylation Kit (Zymo Research Corporation, Irvine, USA). Bisulfite treated DNA was whole genome amplified, enzymatically fragmented and hybridized to the HumanMethylation450 BeadChip. For processing of array-based DNA methylation signals Infinium GenomeStudio software (GenomeStudio 2011.1, methylation module 1.9) was used. Probes with a detection *p* value > 0.01 were excluded from analysis resulting in detection rates of > 99.8 % for all samples. Before starting the differential gene methylation analyses, sample quality was analyzed using scatterplot matrices and density plots of beta values (range: 0 [no methylation], 1 [fully methylated]), which confirmed an overall good quality of the analyzed samples. To identify differentially methylated genes in abdominal fat cells compared to gluteal fat cells, the limma package [19] was used on quantile normalized beta values to compute a moderated t- test. Raw p values were adjusted using the Benjamini-Hochberg procedure [20] and p ≤ 0.01 was used as statistical significant threshold. Differential methylation was calculated by subtracting beta values of abdominal from beta values of gluteal samples.

### Lipomics

For the characterization of triglycerides from abdominal and gluteal adipose tissue samples (n = 14) reversed phase liquid chromatography was combined with the multiple stage mass analysis capability of an ion trap mass spectrometer (RP-HPLC-Ion Trap-MS). To determine the triglyceride distribution, lipid samples (10μl) were solved in methanol (1:10,000) and separated as well as detected by RP-HPLC-Ion Trap-MS. For data analysis, mean values of triglyceride peak areas were calculated. 

To determine fatty acid spectra of abdominal and gluteal adipose tissue (n = 7), lipid samples (5 μl) were dissolved in 100μl Methyl tert-butyl ether (MTBE). After alkaline hydrolysis in sodium hydroxide and methanol (NaOH /Methanol) at 70° C, fatty acids were transferred (at 70° C) into methyl ester derivates with BF3-Methanol (14% Borontrifluoride, 86% Methanol). The final extracts were investigated using gas chromatography combined with a mass spectrometer (GC / MS) on a Rxi-1-MS column. To analyze the fatty acid distribution, mean values of fatty acid methyl ester peak areas were converted into relative values. Normal distribution was tested using the Shapiro-Wilk test and unpaired t tests were performed to test for significance.

### Data deposition

The microarray and methylation data are available in the Gene Expression Omnibus database (http://www.ncbi.nlm.nih.gov/geo/), under accession number GSE47513.

## Results

### Abdominal and gluteal adipose tissues are characterized by a fat depot-specific gene expression pattern

**Figure 1:**
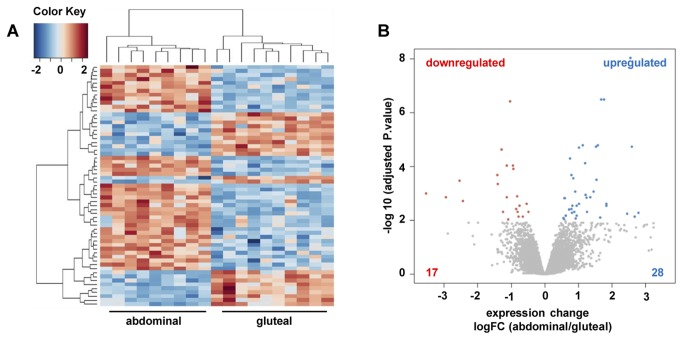
Identification of differentially expressed genes between abdominal and gluteal fat samples. A The heatmap shows 61 gene probes (45 annotated genes) that were differentially expressed between abdominal and gluteal fat depot cells. Columns indicate different donors. B Volcano plot of 32,045 Agilent gene probes. 39 gene probes (28 genes) upregulated in abdominal cells are shown in blue; 22 gene probes (17 genes) downregulated in abdominal cells are shown in red; probes that were not differentially expressed (adj. p value > 0.01) are shown in grey.

To identify differentially expressed genes in abdominal and gluteal adipose tissue, we determined the mRNA transcription profile of 9 abdominal and 10 gluteal adipose tissue sections using Agilent Whole Human Genome Microarrays. Of the 32,045 gene probes analyzed, 31,984 gene probes showed similar array signal intensities in abdominal and gluteal adipose tissue. Expression of 45 genes (corresponding to 61 gene probes) differed significantly (adj. *p* value ≤ 0.01) between abdominal and gluteal adipose tissue samples (Figure 1A). In total, 28 genes (39 gene probes) were significantly upregulated and 17 genes (22 gene probes) were significantly downregulated in abdominal tissue samples compared to gluteal tissue samples as indicated by the volcano plot (Figure 1B). Interestingly, the majority of differentially expressed genes form part of the homeobox gene family including *HOX* gene co-factors such as meis homeobox 1 (*MEIS1*) and meis homeobox 2 (*MEIS2*). We also observed genes that are involved in lipid metabolism such as fibroblast growth factor 10 (*FGF 10*) and phospholipase A2 (*PLA2G2A*), as well as genes which seem to be implicated in metabolic processes such as insulin-like growth factor binding protein 5 (*IGFBP5*). Together, these findings demonstrate that abdominal and gluteal adipose tissues show distinct gene expression profiles that might account for functional differences. 

### Epigenetic mechanisms are involved in depot-specific gene regulation

**Figure 2:**
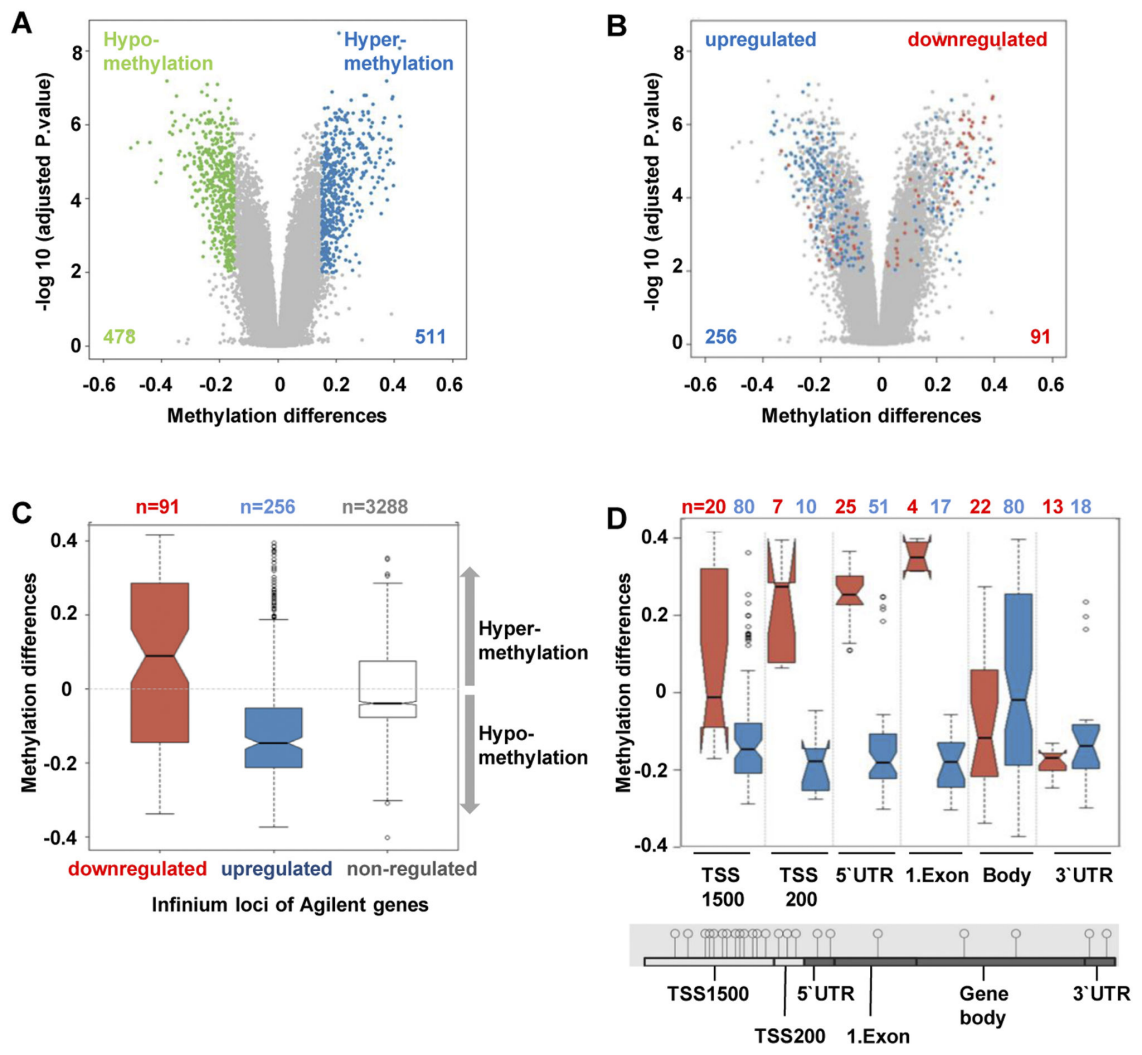
DNA methylation correlates with gene expression in abdominal and gluteal fat samples. A Mean methylation differences between abdominal and gluteal fat depot cells were plotted against Benjamini-Hochberg adjusted p values. Green indicates hypomethylation, blue indicates hypermethylation and grey indicates non-significant methylation differences; adjusted p value for significance: ≤ 0.01. B Volcano plot of log of Benjamini-Hochberg adjusted p values versus mean methylation differences between abdominal and gluteal fat samples. Red indicates CpGs corresponding to genes showing lower expression in abdominal cells (down-regulated); blue indicates CpGs corresponding to genes showing higher expression in abdominal cells (up-regulated), grey indicates non-significant differences between gene probes; adjusted p value ≤ 0.01. C,D The boxplots show the distribution and mean methylation changes of probes of down-regulated (red), up-regulated (blue) and non-regulated (grey) genes for all gene-associated CpGs (**C**) and for CpG loci classified according to their genomic locations (**D**). The numbers above indicate the numbers of probes in each category. The horizontal black lines denote medians, notches the standard errors, boxes the interquartile range, and whiskers the 2.5th and 97.5th percentiles. Mann-Whitney-U-tests indicate highly significant differences (p = 9.03 × 10-9) between methylation differences of up- and down-regulated genes.

To investigate whether differential expression of these genes might be regulated by epigenetic mechanisms we additionally determined global methylation patterns of 6 abdominal and 6 gluteal adipose tissues. Using the *HumanMethylation450 Bead Chip* (Illumina), which allows to interrogate the genome-wide methylation status of more than 450,000 CpG dinucleotides per sample, we identified differentially methylated genes in abdominal versus gluteal fat depot samples. Methylation analyses revealed a set of 223 significantly differentially methylated genes (represented by 989 CpG dinucleotides) between abdominal and gluteal fat samples. 121 genes (511 CpGs) were hypermethylated (differential methylation > 0.15) in abdominal versus gluteal fat samples and 102 genes (corresponding to 478 CpGs) were hypomethylated (differential methylation < -0.15) as indicated in the Volcano plot (Figure 2A). To investigate the correlation between differential expression and methylation, the Agilent expression data were mapped to methylation probes present on the *HumanMethylation450 Bead Chip* (Illumina). A total of 25 genes (corresponding to 347 CpGs) showed significant differences in gene expression and DNA methylation, suggesting that a great proportion (55 %) of differentially expressed genes might be regulated by epigenetic mechanisms.

To investigate the correlation between DNA methylation and gene expression in more detail, we separated differentially expressed genes in 16 upregulated genes (256 CpG loci) and 9 downregulated genes (91 CpG loci) and analyzed the methylation differences at the corresponding loci. Volcano plots in Figure 2B illustrate significantly differentially methylated CpGs according to their association with upregulated or downregulated genes in abdominal and gluteal fatty tissue samples. We found that CpGs corresponding to genes showing higher expression in abdominal cells (256 CpG dinucleotides, shown in blue, here referred to as “upregulated genes”) are generally associated with hypomethylation whereas CpGs corresponding to genes showing lower expression in abdominal cells compared to gluteal cells (91 CpG dinucleotides, shown in red, here referred to as “downregulated genes”) are more associated with hypermethylation. To quantify methylation differences between abdominal and gluteal fat samples, the distribution of methylation differences in upregulated, downregulated and non-regulated genes was visualized with boxplots. The boxplot in Figure 2C clearly shows that genes upregulated in abdominal cells show decreased CpG methylation levels whereas genes downregulated in abdominal cells are more associated with CpG hypermethylation. Mann-Whitney-U-tests indicate highly significant differences (p = 9.03 × 10-9). To analyze the correlation of methylation status and expression levels more extensively, the methylation differences of the 347 Infinium probes (256 CpG dinucleotides for upregulated and 91 CpG dinucleotides for downregulated genes) were plotted according to their location in different genomic regions. Genes upregulated in abdominal tissue samples generally showed reduced methylation levels in regions close to the transcription start site (TSS200, 5´-UTR) and the first exon of genes (Figure 2D) whereas genes downregulated in abdominal cells showed a clear trend towards hypermethylation in these regions. For the TSS1500 and gene body regions as well as for the 3´-UTR no definite conclusion could be drawn because of the spread of the data. Furthermore we found that annotated differentially methylated regions (DMRs) as well as DNAse I hypersensitive sites (DHSI) and enhancer regions showed significant methylation differences between abdominal and gluteal tissues indicating that methylation changes in these regions might also be of functional relevance for depot-specific gene expression (Figure S2). In summary, hypermethylation of promoter regions was generally associated with transcriptional silencing whereas hypomethylation appeared to allow active transcription suggesting that epigenetic mechanisms are involved in the depot-specific regulation of a defined set of genes.

### Fat depot-specific expression and methylation are associated with developmental processes

**Figure 3:**
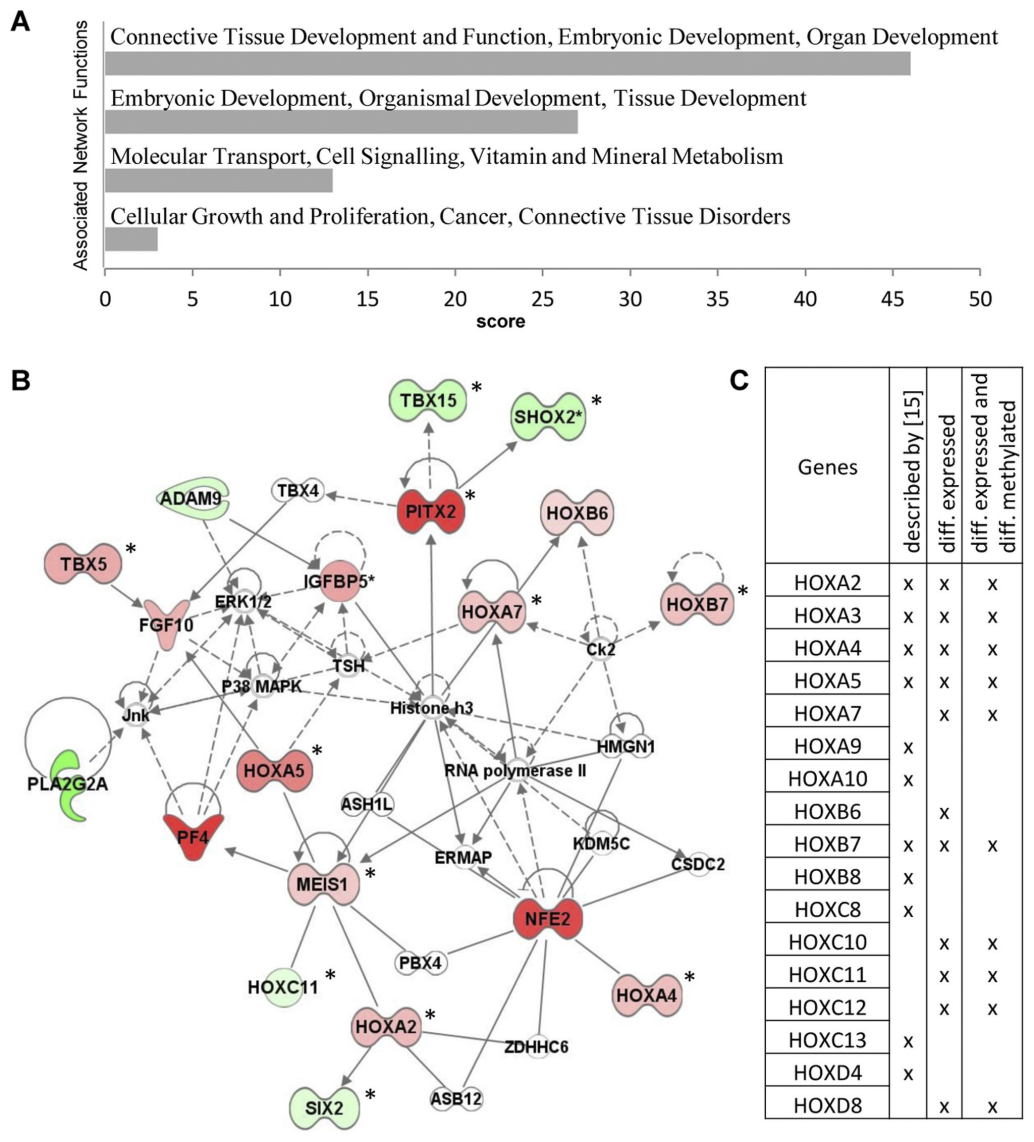
Pathway analysis of differentially expressed genes in abdominal fat samples compared to gluteal fat samples. A Top associated networks for differentially expressed genes in abdominal cells derived from IPA (Ingenuity pathway analysis). B Top network built by differentially expressed genes in abdominal fat cells compared to gluteal fat cells. Red color indicates upregulated genes in abdominal cells, green color downregulated genes in abdominal cells. Genes marked with asterisks showed differential methylation between both depots. ADAM9, ADAM metallopeptidase domain 9; FGF10, fibroblast growth factor 10; HOXA2, homeobox A2; HOXA4, homeobox A4; HOXA5, homeobox A5; HOXA7, homeobox A7; HOXB6, homeobox B6; HOXB7, homeobox B7; HOXC11, homeobox C11; IGFBP5, insulin-like growth factor binding protein 5; MEIS1, Meis homeobox 1; NFE2, nuclear factor (erythroid-derived 2); PF4, platelet factor 4; PITX2, paired-like homeodomain; *PLA2G2A*, phospholipase A2, group IIA; *SHOX2*, short stature homeobox 2; SIX2, SIX homeobox 2; TBX5, T-box 5; TBX15, T-box 15. C Comparative illustration of *HOX* genes that (1) have been previously described as differentially expressed in gluteal and abdominal depots [14], (2) *HOX* genes, that were found to be differentially expressed in our study and (3) *HOX* genes that showed differential expression and differential methylation in our study.

To investigate the biological relevance of genes which were differentially expressed or genes which were differentially expressed and differentially methylated between abdominal and gluteal depots (in total 45 genes), network and pathway analysis were prepared by Ingenuity Pathway Analysis (IPA; Ingenuity Pathway Analysis, Analysis Systems). These analyses revealed an enrichment of functional categories associated with development (Figure 3A) with the top-score network “Connective Tissue Development and Function, Embryonic Development and Organ Development” which is drawn in Figure 3B. Recently, Karastergiou et al. (2012) showed that developmental factors, especially *HOX* genes, play an important role in defining depot-specific functional characteristics of adipose tissue in both sexes [14]. In line with these data, we observed a specific set of homeobox genes in women, that was differentially expressed between abdominal and gluteal adipose tissue. Remarkably, we identified the same *HOX* clusters as described by Karastergiou et al. (2012) [14] and a number of identical *HOX* genes such as *HOXA2*, *HOXA3*, *HOXA4* and *HOXA5* (Figure 3C). Additionally we identified *HOXB6* and *HOXD8* which showed increased expression in the abdominal depot and *HOXC10*, *HOXC11* and *HOXC12* with an increased expression in the gluteal depot (Figure 3C). Importantly, the majority of these *HOX* genes showed depot-specific methylation differences (Figure 3B, genes marked with asterisks and Figure 3C) suggesting an involvement of epigenetic mechanisms in the regulation of adipose tissue specific developmental programs. 

**Figure 4:**
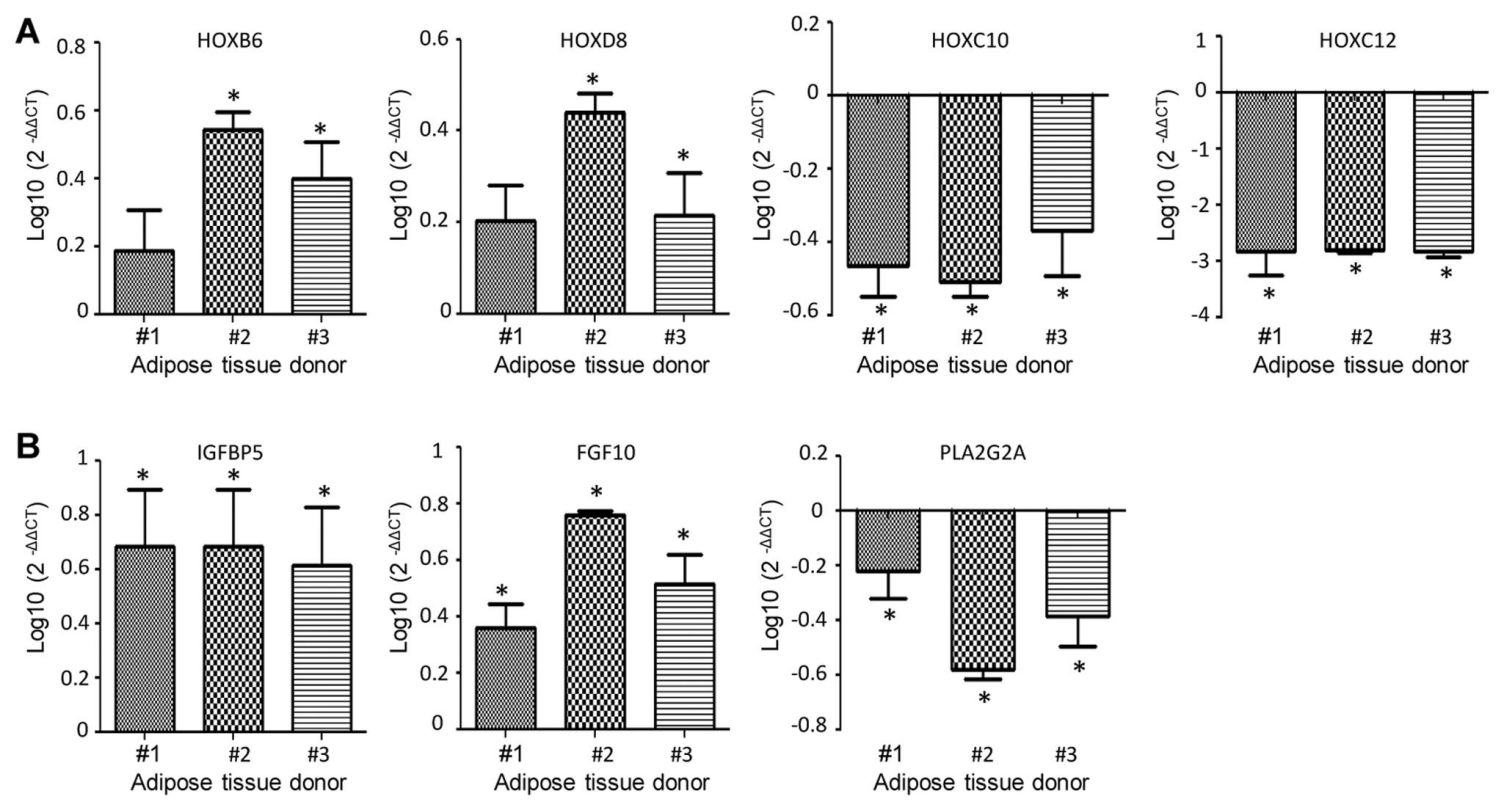
Quantitative RT-PCR of genes with depot-specific gene expression or genes with depot-specific gene expression and depot-specific methylation. Asterisks indicate a significance level of 0.05 (alpha). A,B An independent validation series of samples (abdominal n = 3, gluteal n = 3) was used to confirm the differential expression detected by genome-wide analysis. Target gene expression was normalized to gluteal and GAPDH was used as a reference. Data are presented as mean + SEM. (**A**) Homeobox B6 (HOXB6), homeobox D8 (HOXD8), homeobox C10 (HOXC10), homeobox C12 (HOXC12), (**B**) Insulin-like growth factor binding protein 5 (IGFBP5), Fibroblast growth factor 10 (FGF10) and PhospholipaseA2, group2 (PLA2G2A).

In addition to genes involved in developmental processes, we identified differentially expressed genes which might play a role in metabolic-related diseases or are involved in lipid metabolism such as phospholipase A2, group 2A (*PLA2G2A*), Fibroblast growth factor 10 (*FGF10*) and insulin-like growth factor binding protein 5 (*IGFBP5*). For *IGFBP5*, differential expression was associated with depot-specific differences in methylation pattern. To further confirm our gene expression data, we validated depot-specific expression of selected *HOX* genes, that had not previously been described to be differentially expressed between abdominal and gluteal depots as well as differential expression of genes involved in fat cell metabolism. Using qRT-PCR analysis of an independent sample set (n = 3) differential expression could be successfully validated (Figure 4). Technical validation of the expression data using the same samples as in the original microarray experiment (n = 5) is shown in the supplemental data (Figure S3). In summary, our data provide evidence for the involvement of epigenetic mechanisms in fat depot-specific regulation of developmental processes that might account for functional differences between both depots.

### Triglycerides and fatty acids are more saturated in abdominal adipose tissue

**Figure 5:**
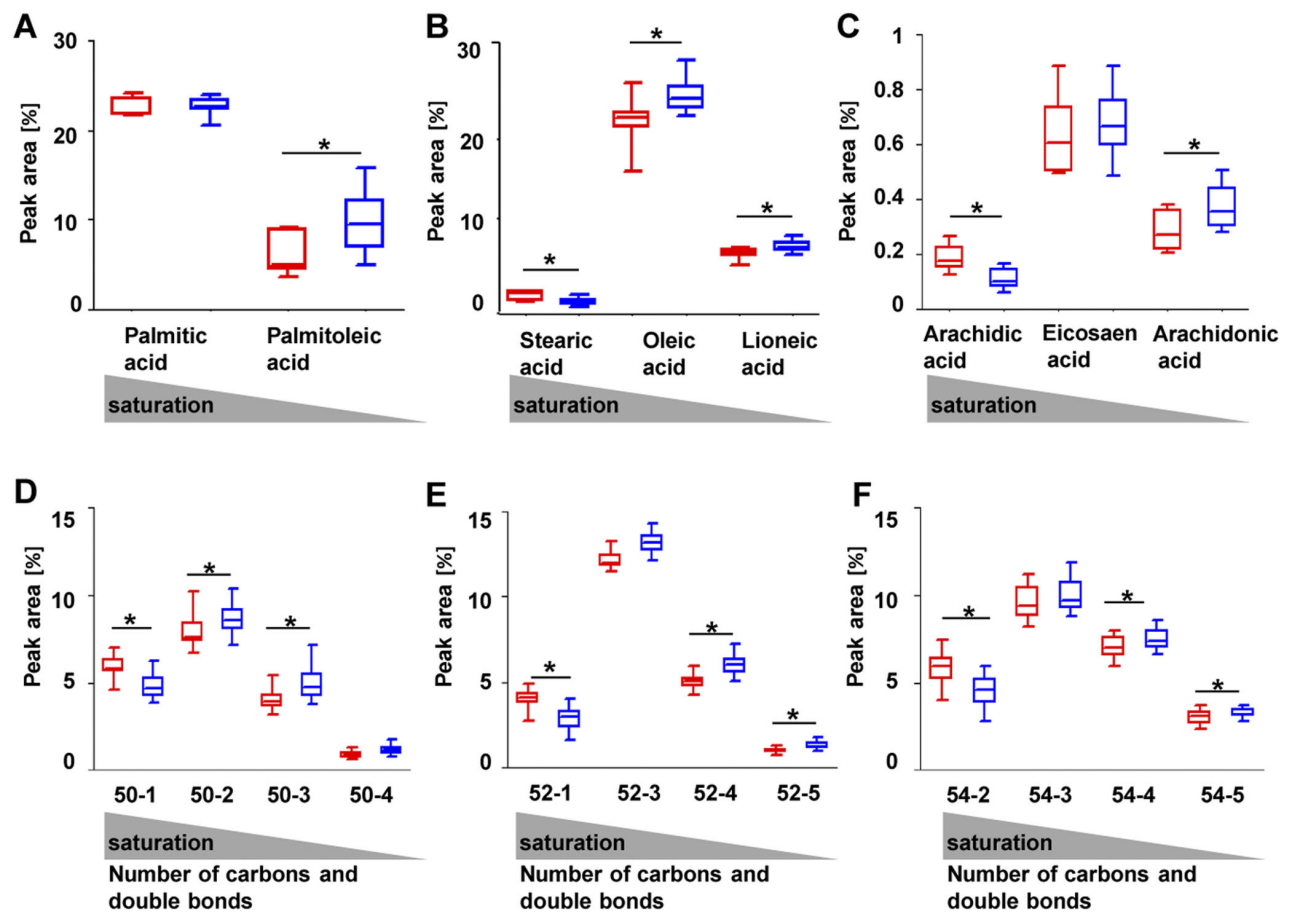
RP-HPLC-MS (Reverse phase high-performance liquid chromatography-mass spectrometry) of abdominal and gluteal fat samples. Red colors represent abdominal depots, blue colors represent gluteal depots. A,B,C Boxplots show fatty acid saturation of abdominal and gluteal adipose tissue. D,E,F Boxplots show triglyceride saturation of abdominal and gluteal adipose tissue. First numbers indicate the number of atomic carbons, second numbers indicate the number of double bonds (e.g. 50-1; 50 atomic carbons (50 C), one double bond).

Having observed differentially expressed genes which are involved in fatty acid metabolism we compared the composition of lipids between abdominal and gluteal depots. Therefore, we measured the triglyceride and fatty acid distribution by Reverse Phase High-Performance liquid chromatography-mass spectrometry (RP-HPLC-MS). Our results indicate significant differences in fatty acids and triglyceride saturation in both depots as shown in Figure 5. The boxplots display different types of fatty acids with different states of saturation. Palmitic acid (Figure 5A), Stearic acid (Figure 5B) and Arachidic acid (Figure 5C) represent saturated fatty acids that are more enriched in abdominal adipose tissue compared to gluteal adipose tissue. Palmitoleic acid, Oleic acid and Lioneic acid, as well as Arachidonic acid represent more unsaturated fatty acids that were detected with higher ratios in gluteal adipose tissue compared to abdominal adipose tissue. Similar results were observed by analyzing of triglyceride saturation in both depots. In detail, saturated triglycerides were detected with higher frequency in abdominal adipose tissue whereas more unsaturated triglycerides showed a higher enrichment in gluteal adipose tissue (Figure 5D-F). To summarize, fatty acids and triglycerides were significantly more saturated in abdominal adipose tissue compared to gluteal adipose tissue.

## Discussion

Adipose tissue is distributed in abdominal and gluteal fat depots that comprise a variety of functional differences. These differences can be associated with different risks for metabolic-related disorders with excessive abdominal adipose tissue being more associated with metabolic disorders compared to gluteal adipose tissue. Therefore, we aimed to characterize adipose tissue samples of abdominal and gluteal areas at the molecular level to understand its heterogeneous functions and to develop new strategies to treat obesity or metabolic syndrome-associated complications. 

We investigated depot-specific differences in lipid compositions and observed that triglycerides and fatty acids were more saturated in abdominal adipose tissue compared to gluteal adipose tissue. Interestingly, using genome-wide expression analysis we identified differentially expressed genes that are involved in lipid metabolism such as phospholipase A2, group 2A (*PLA2G2A*) and fibroblast growth factor 10 (*FGF10*). Iyer et al. (2012) showed that *PLA2G2A* is an important mediator in the cross talk between immune and metabolic systems in adipose tissue with relevance for lipid and energy homeostasis as well as for metabolic and cardiovascular function [21]. For *FGF10*, which showed increased expression in the abdominal depot, a regulatory function in adipose development and metabolism has been reported [22]. However, further experiments will be required to clarify the specific effects of differential *PLA2G2A* and *FGF10* regulation on lipid metabolism. 

Furthermore, we observed *IGFBP-5*, which shows differential expression and methylation in abdominal versus gluteal depots. *IGFBPs* are well-known critical regulators of the mitogenic activity of insulin-like growth factors (IGFs). Of note, abnormal expression of other *IGFBP* such as *IGFBP-1* and *IGFBP-2* was detected in different states of metabolic disorders where reduced serum levels of both proteins were associated with cardiovascular risk factors [23,24]. Therefore, genes belonging to the *IGFBP* family have been considered as early markers for the development of metabolic syndrome [24]. Consequences of higher *IGFBP-5* expression levels for metabolic complications have not been analyzed in detail yet; however, these findings suggest a possible involvement of *IGFBP-5* in the development of metabolic disorders in abdominal adipose tissue.

In addition to the previously discussed genes, we observed a specific set of homeobox genes, that was differentially expressed between abdominal and gluteal adipose tissue. Interestingly, multiple anterior *HOX* genes were expressed at higher levels in abdominal than in gluteal adipose tissue. In addition to their function as transcription factors during embryonic development, *HOX* genes are also expressed in adult tissues, where they regulate differentiation processes and thereby determine functional characteristics of these tissues [25]. It has been further reported that the *HOX* gene network is involved in transcriptional regulation of human adipogenesis in vivo [26] and that genes involved in embryonic development potentially play important roles in adipocyte development and fat distribution [27]. Furthermore, Dankel et al. (2010) observed a switch from stress response to increased expression of homeobox transcription factors after fat loss which indicates potential roles for homeobox genes in regulating adipose tissue function [28]. Our data therefore support the hypothesis that developmental genes are implicated in defining depot-specific characteristics and functions of adult human adipose tissue. 

Furthermore, our data indicate that DNA methylation is involved in the regulation of depot-specific gene expression. A total of 989 CpGs showed a significant differential methylation (methylation change > 0.15) in abdominal depots compared to gluteal depots. Roughly one third of these loci (347 CpGs) seemed to affect gene expression. We also analyzed the overall DNA methylation pattern in more detail plotting the 989 CpGs according to their location in different genomic regions (Figure S4). Interestingly, a large proportion of these differentially methylated gene loci were located in the gene body region (48 %) and just a small proportion of them could be associated with gene expression changes. The role of DNA methylation in gene bodies and its association with gene expression phenotypes is not well understood yet and appears to be subject of complex regulation mechanisms. Several groups showed a positive correlation between active gene expression and gene body methylation whereas others did not observe any clear relationship [29,30]. Otherwise, it has been consistently shown that DNA methylation changes at promoter regions are strongly associated with gene expression changes. Consistent with the general acceptance, the majority of differentially methylated CpGs located within the promoter region (TSS1500, TS200; 117 of 138 CpGs) showed a strong association between hypomethylation and active gene expression as well as between hypermethylation and lower gene expression levels suggesting that methylation differences at these genomic locations are of relevance for gene regulation, also in adipose tissue. Interestingly, the majority of the differentially methylated genes in abdominal fat cells form also part of the homeobox gene family. Methylation of specific *HOX* genes has been described in the context of cancer; however, no genome-wide comparative DNA methylation analysis of depot-specific adipose tissue has been conducted yet. For the first time our data show, that DNA methylation differences at *HOX* and other developmental genes might be involved in differential gene regulation in abdominal and gluteal adipose tissues. Also for diseases such as obesity, epigenetics has emerged as a very important determinant. For example, Wang et al. (2010) showed that obesity is associated with methylation changes in blood leukocyte DNA [31].

In summary, our data provide first evidence that epigenetic mechanisms are involved in the regulation of developmental processes specifying depot-specific characteristics. These depot-specific signatures strongly suggest different functional roles for abdominal and gluteal subcutaneous adipose tissue and provide new insights into the role of epigenetics in regulating depot-specific characteristics. Further investigations on the involvement of epigenetic mechanisms in the development of metabolic disorders will show, whether epigenetic therapy approaches might be useful as a treatment strategy for metabolic diseases.

## Supporting Information

Figure S1 **Descriptive table showing detailed characteristics (age and BMI) of all volunteers and samples used for analyses (gene expression, validation of gene expression, DNA methylation profiling as well as triglyceride and fatty acid composition).**
(TIF)

Figure S2 **Methylation differences in specific genomic regions such as differentially methylated regions (DMRs), DNAse I hypersensitive sites and enhancers.**
(TIF)

Figure S3 **Technical validation of gene expression array data by quantitative RT-PCR of selected genes.**
(TIF)

Figure S4 **Methylation changes between abdominal and gluteal adipose tissue at 989 differentially methylated CpG loci (Log FC > 0.15) classified according to their genomic locations (TSS1500, TSS200, 5´UTR, 1^st^ Exon, Body, 3´UTR).**
(TIF)
