# Supplementary figures and images for: Epigenetic Regulation of Depot-Specific Gene Expression in Adipose Tissue
Source: PLoS One. 2013 Dec 5;8(12):e82516. doi: 10.1371/journal.pone.0082516 (PMC3855576; doi:10.1371/journal.pone.0082516)

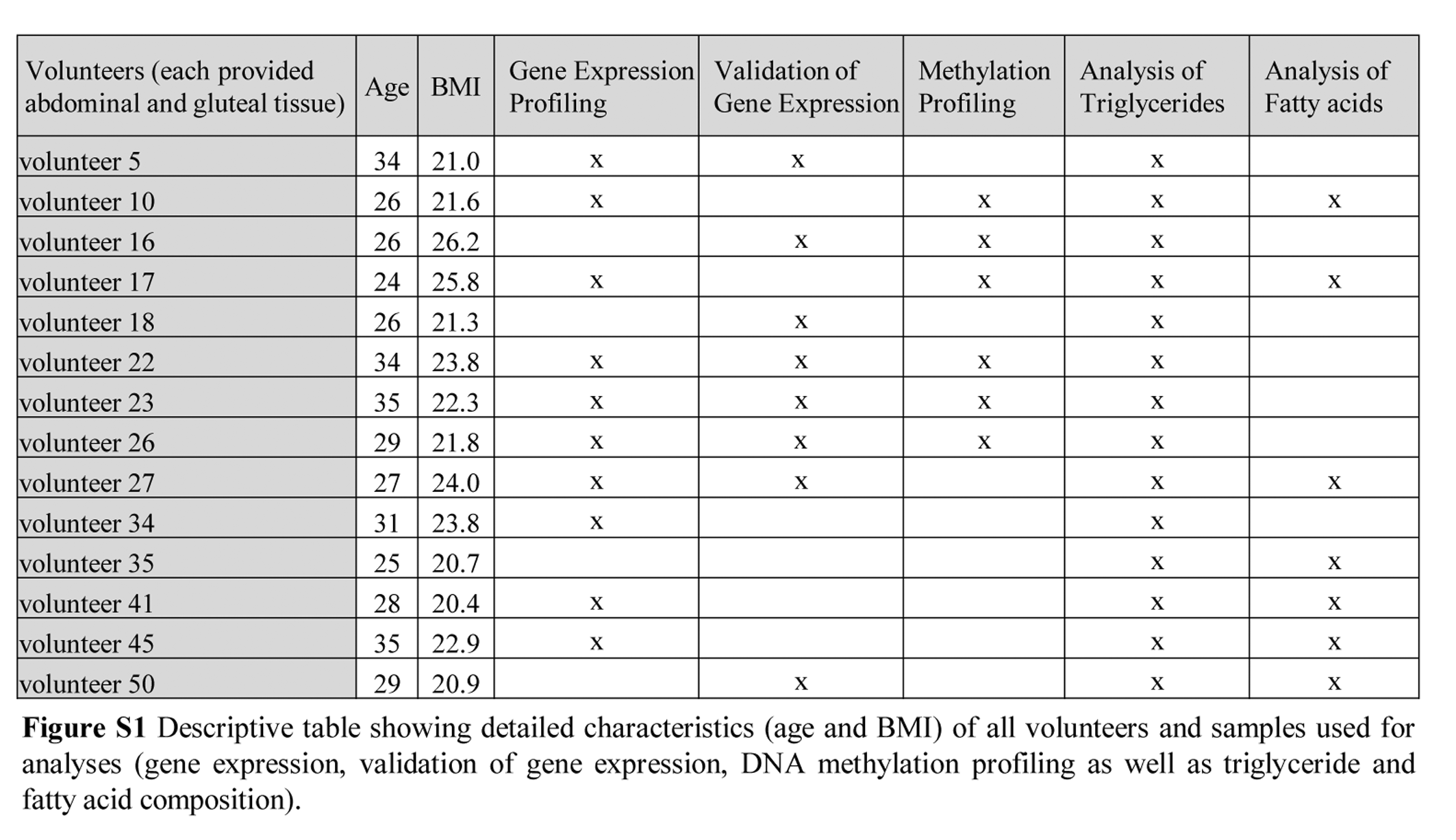

Supplement: Figure S1 — Descriptive table showing detailed characteristics (age and BMI) of all volunteers and samples used for analyses (gene expression, validation of gene expression, DNA methylation profiling as well as triglyceride and fatty acid composition). (TIF) [file pone.0082516.s001.tif]

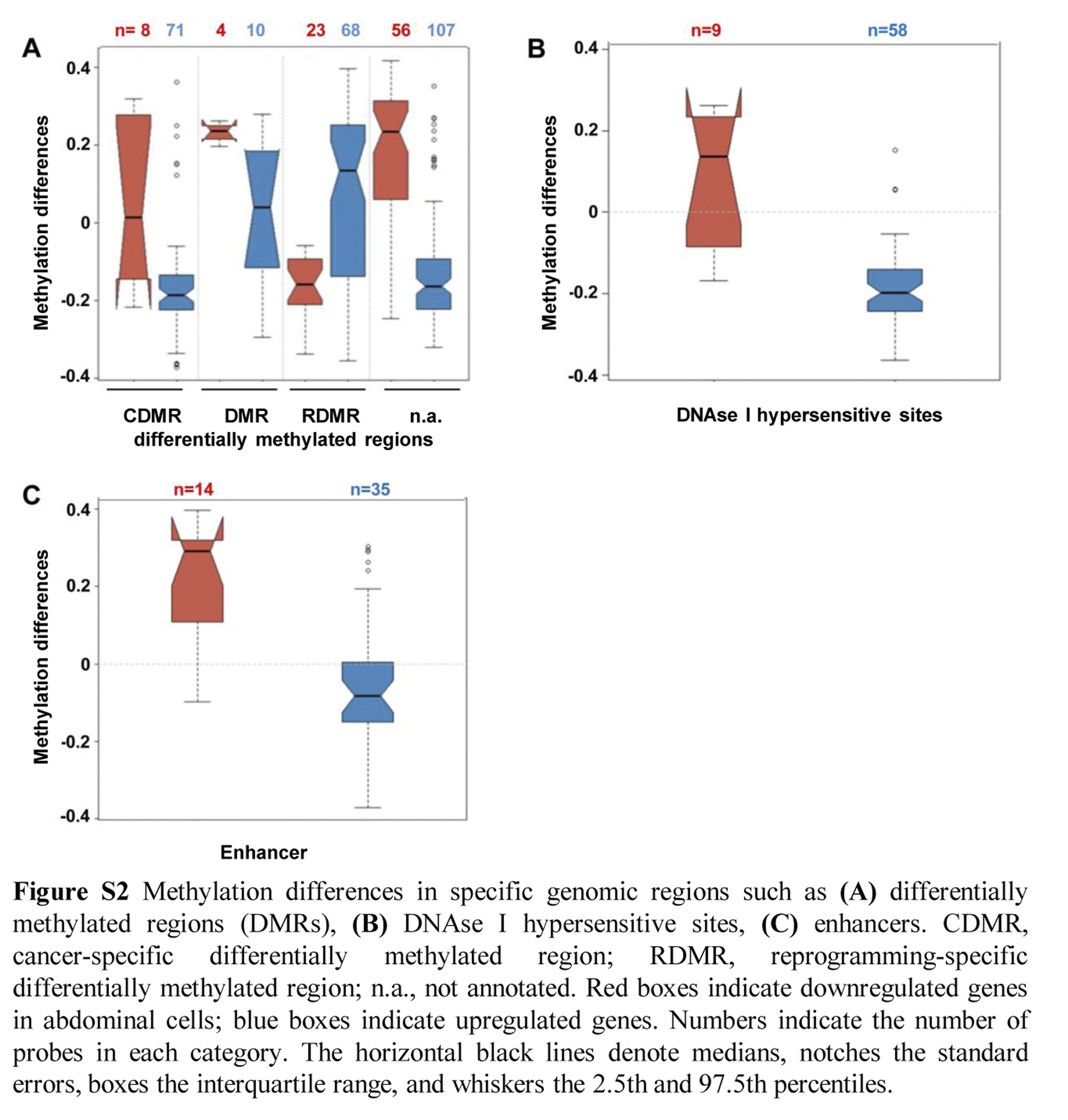

Supplement: Figure S2 — Methylation differences in specific genomic regions such as differentially methylated regions (DMRs), DNAse I hypersensitive sites and enhancers. (TIF) [file pone.0082516.s002.tif]

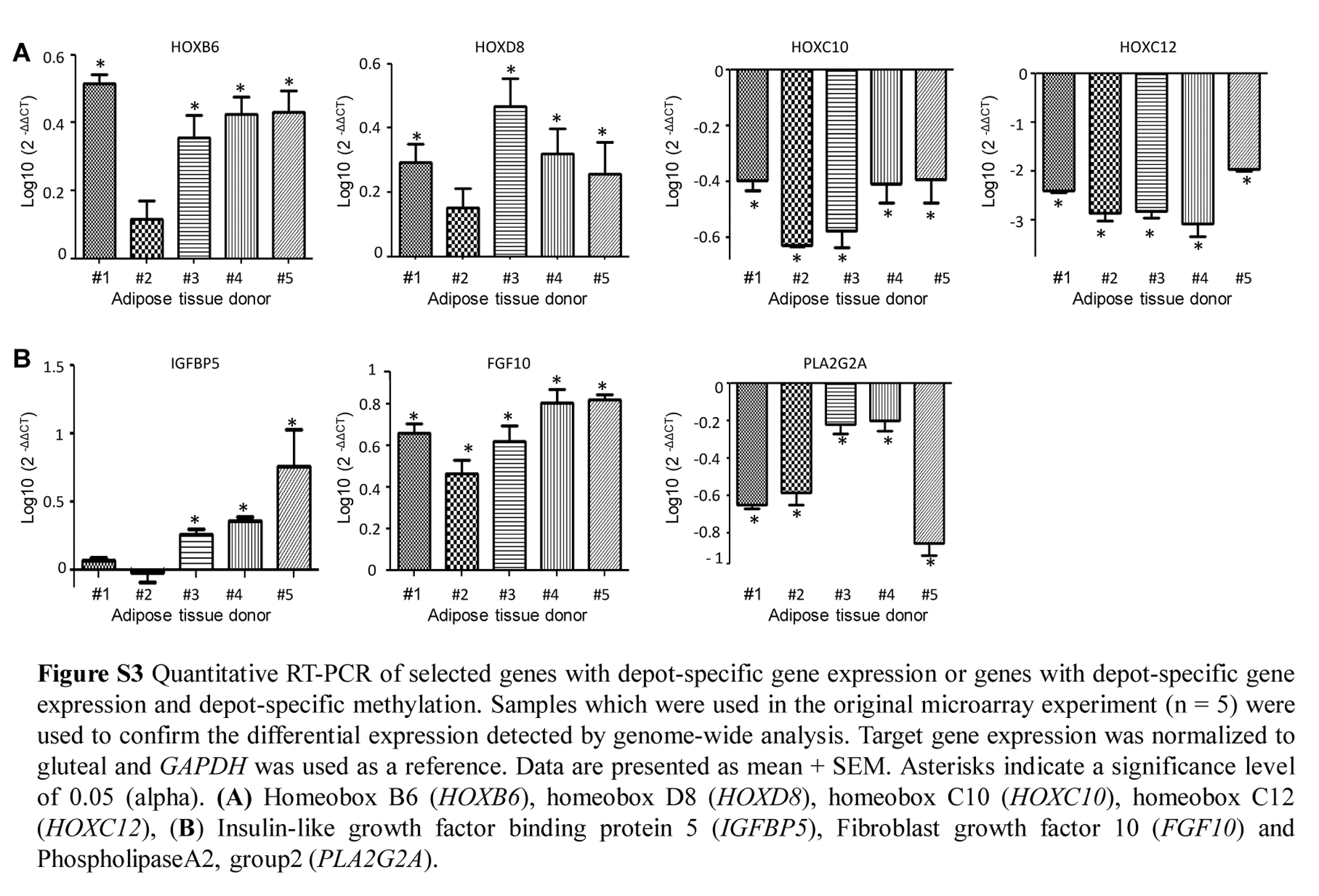

Supplement: Figure S3 — Technical validation of gene expression array data by quantitative RT-PCR of selected genes. (TIF) [file pone.0082516.s003.tif]

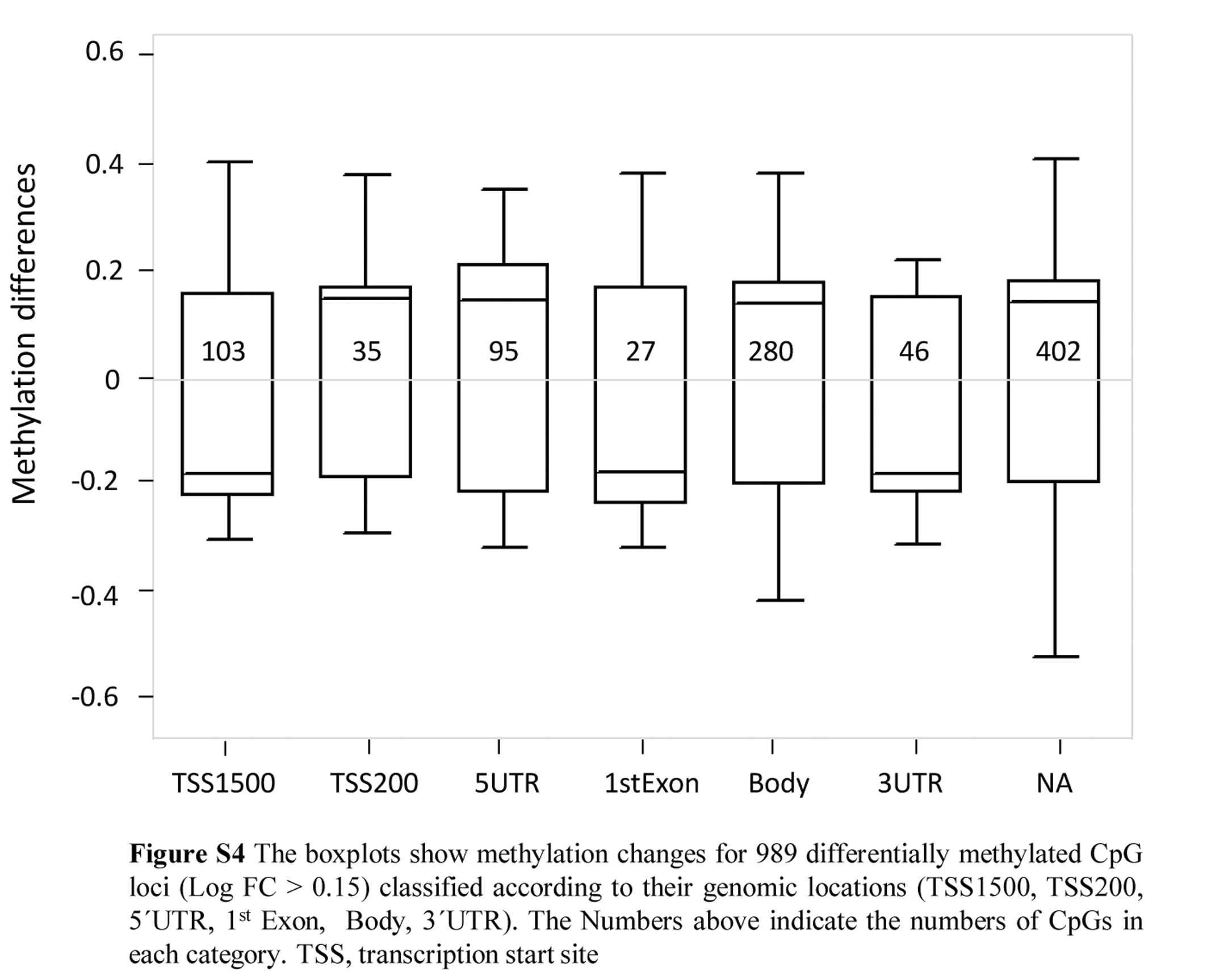

Supplement: Figure S4 — Methylation changes between abdominal and gluteal adipose tissue at 989 differentially methylated CpG loci (Log FC > 0.15) classified according to their genomic locations (TSS1500, TSS200, 5´UTR, 1st Exon, Body, 3´UTR). (TIF) [file pone.0082516.s004.tif]
